# Determination of Genetic Effects of *LIPK* and *LIPJ* Genes on Milk Fatty Acids in Dairy Cattle

**DOI:** 10.3390/genes10020086

**Published:** 2019-01-28

**Authors:** Lijun Shi, Bo Han, Lin Liu, Xiaoqing Lv, Zhu Ma, Cong Li, Lingna Xu, Yanhua Li, Feng Zhao, Yuze Yang, Dongxiao Sun

**Affiliations:** 1 Department of Animal Genetics, Breeding and Reproduction, College of Animal Science and Technology, Key Laboratory of Animal Genetics, Breeding and Reproduction of Ministry of Agriculture and Rural Affairs, National Engineering Laboratory for Animal Breeding, China Agricultural University, Beijing 100193, China; shilijun_1111@126.com (L.S.); bohan@cau.edu.cn (B.H.); li10020902@163.com (C.L.); Lingna_na_Xu@163.com (L.X.); 18800051836@163.com (Y.L.); 2 Beijing Dairy Cattle Center, Qinghe’nanzhen Deshengmenwai Street, Chaoyang District, Beijing 100192, China; liulin@bdcc.com.cn (L.L.); 13810787287@139.com (X.L.); 13810063288@163.com (Z.M.); zhaofeng@bdcc.com.cn (F.Z.); 3 Beijing General Station of Animal Husbandry, N0.96 Huizhongsi, Yayun Village, Chaoyang District, Beijing 100101, China; yyz84929056@126.com

**Keywords:** lipase family member K, lipase family member J, genetic effects, milk fatty acids, dairy cattle

## Abstract

In our previous genome-wide association study (GWAS) on milk fatty acids (FAs) in Chinese Holstein, we discovered 83 genome-wide significant single nucleotide polymorphisms (SNPs) associated with milk FAs. Two of them were close to lipase family member K (*LIPK*) and lipase family member J (*LIPJ*), respectively. Hence, this study is a follow-up to verify whether the *LIPK* and *LIPJ* have significant genetic effects on milk FAs in dairy cattle. By re-sequencing the entire exons, and 3 kb of 5′ and 3′ flanking regions, two and seven SNPs were identified in *LIPK* and *LIPJ*, respectively, including a novel SNP, ss158213049726. With the Haploview 4.1 software, we found that five of the SNPs in *LIPJ* formed a haplotype block (D′ = 0.96 ~ 1.00). Single-locus association analyses revealed that each SNP in *LIPK* and *LIPJ* was significantly associated with at least one milk FA (*p* = < 1.00 × 10^−4^ ~ 4.88 × 10^−2^), and the haplotype-based association analyses showed significant genetic effects on nine milk FAs (*p* = < 1.00 × 10^−4^ ~ 3.98 × 10^−2^). Out of these SNPs, the missense mutation in *LIPK* gene, rs42774527, could change the protein secondary structure and function predicted by SOPMA, SIFT, and PROVEAN softwares. With the Genomatix software, we predicted that two SNPs, rs110322221 in *LIPK* and rs211373799 in *LIPJ*, altered the transcription factors binding sites (TFBSs), indicating their potential regulation on promoter activity of the genes. Furthermore, we found that both *LIPK* and *LIPJ* had relatively high expressions in the mammary gland. In conclusion, our research is the first to demonstrate that *LIPK* and *LIPJ* genes have significant associations with milk FAs, and the identified SNPs might be served as genetic markers to optimize breeding programs for milk FAs in dairy cattle. This research deserves in-depth verification.

## 1. Introduction

In the dairy industry, milk production traits are the most important factors when considering breeding goals, especially milk composition, milk protein and fat, which account for main focus of genetic improvement programs designed to increase the productivity of dairy cattle. Fatty acid, as found in milk fat, is rich in saturated fatty acids (SFAs) [1]. Diets containing too much SFA are strongly correlated with the development of nonalcoholic fatty liver disease (NAFLD) [2]. Furthermore, NAFLD has been shown to threaten human health through, with it leading to obesity, dyslipidemia, and hypertension [3,4]. Therefore, it will be desirable to enrich dairy products with unsaturated fatty acids (UFAs) to promote the health of consumers [1]. Milk fatty acid (FA) is a kind of quantitative trait that is controlled by many factors including genetic and environmental factors. Supplementing dairy cow diets with plant oils has been shown to lower milk SFA concentrations, and increase milk trans-fatty acids [5]. During rumen fermentation, the specific FAs, such as *trans-10* and *cis-12*, can down-regulate the expression of key lipogenic genes involved in milk fatty synthesis, which also can indirectly regulate milk FAs [6]. In regard to genetics, previous studies have reported that the estimates of heritability for SFA and UFA were 0.14 to 0.33 and 0.08 to 0.29, respectively, in Holstein cattle [7,8,9,10,11]. For milk FAs in dairy cattle, some promising candidate genes were identified by genome-wide association studies (GWAS) [12,13,14], and their genetic effects were further confirmed through association analyses [15,16,17].

In our previous GWAS on milk FAs in Chinese Holstein cattle [13], we found two single nucleotide polymorphisms (SNPs) close to the *lipase family member K* (*LIPK*, Chr26: 10409542 ~ 10450528) and lipase family member J (*LIPJ*, Chr26: 10211395 ~ 10248584), respectively. The SNP, ARS-BFGL-NGS-21794 (rs110933619), located upstream of the *LIPK* gene with a distance of 35.29 kb, was strongly associated with C14:1 (*p* = 9.57 × 10^−8^) and C14index (*p* = 4.20 × 10^−10^); and the SNP, BTA-61921-no-rs (rs209033376), in the downstream of *LIPJ* gene with a distance of 7.41 kb, showed significant associations with the C14:1 (*p* = 1.42 × 10^−5^) and C14index(*p* = 6.99 × 10^−8^). Hence, *LIPK* and *LIPJ* were considered as promising candidate genes for milk FAs.

*LIPK* and *LIPJ* genes are two members of the lipase family. Lipases are hydrolytic enzymes that catalyze the hydrolysis and synthesis of a variety of acylglycerols at the interface of lipid and water [18]. In humans, the *LIPK* and *LIPJ* encoded epidermal lipases and testis lipase, respectively [19]. A study showed that *LIPK* and *LIPJ* genes are the predominant lipases/esterases used by mycobacterium tuberculosis for the storage and degradation of host-derived fat [20]. According to the Ensembl database (http://asia.ensembl.org/index.html), *LIPK* and *LIPJ* genes are related to lipase activity, and are involved in the lipid catabolic process.

In addition, Li et al. identified a large region (10.00 to 27.94 Mb) on BTA26 affecting C14:1 and C14index through the GWAS based on the combined populations including 784 Chinese Holstein used in our previous GWAS [13] and 371 Danish Holstein [14], which included *LIPK*, *LIPJ*, and *SCD* genes. The *LIPK* and *LIPJ* genes were in the upstream of *SCD* with a distance of 10.69 and 10.89 Mb, respectively. We have verified the significant genetic associations of *SCD* with the milk FAs [17]. Therefore, the purpose of this study was to validate the genetic effects of *LIPK* and *LIPJ* with milk FAs in dairy cattle. Furthermore, we estimated the linkage disequilibrium (LD) among SNPs of *LIPK*, *LIPJ*, and *SCD* to identify whether their genetic associations were due to the effects of *SCD*.

## 2. Materials and Methods

### 2.1. Animal Population and Phenotypic Data

In this study, we used a total of 1065 Chinese Holstein cows from 44 sires, which is a different population from those used in our previous GWAS. The cows were from 23 dairy farms belonging to the Sanyuanlvhe Dairy Farming Center (Beijing, China), where the Dairy Herd Improvement system (DHI) has been carried out since 1999. In the dairy industry, DHI means a monthly routine standard milk production measurement system that is used to test the phenotypic values of milk protein percentage, fat percentage, and somatic cell count etc. using individual milk samples of dairy cows. During November to December of 2014, 50 mL milk samples were collected from the 1065 cows to be used for measuring DHI in the Beijing Dairy Cattle Center (www.bdcc.com.cn). After DHI measuring, we used 2 ml milk samples to measure the milk FAs by gas chromatography.

The detailed descriptions of the measuring method used for milk FAs have previously been discussed [13]. In this study, 16 single milk FAs, including C6:0 (caproic acid), C8:0 (caprylic acid), C10:0 (capric acid), C11:0 (undecanoic acid), C12:0 (lauric acid), C13:0 (tridecanoic acid), C14:0 (myristic acid), C14:1 (myristoleic acid), C15:0 (pentadecanoic acid), C16:0 (palmitic acid), C16:1 (palmitoleic acid), C17:0 (margaric acid), C17:1 (heptadecenoic acid), C18:0 (stearic acid), C18:1cis-9 (cis-olein), and C20:0 (arachidic acid), were directly measured; five milk FAs (C14index, C16index, C17inex, C18index and total index) were calculate based on the formulas:

$\frac{\mathrm{cis}-9\;\mathrm{unsaturated}}{\mathrm{cis}-9\;\mathrm{unsaturated}+\mathrm{saturated}}*100$

[21]; and SFA, UFA, and SFA/UFA, were calculated according to the groups of SFA (C6:0, C8:0, C10:0, C11:0, C12:0, C13:0, C14:0, C15:0, C16:0, C17:0, C18:0, and C20:0), and UFA (C14:1, C16:1, C17:1, and C18:1cis-9). The summary of the phenotype values is shown in Table S1.

### 2.2. SNPs Identification and Genotyping

Based on the genomic sequences of *LIPK* (GenBank accession number: AC_000183.1) and *LIPJ* (GenBank accession number: AC_000183.1), we respectively designed 21 and 23 pairs of primers using the Primer 3 (http://bioinfo.ut.ee/primer3-0.4.0/) in the entire exons with their partial adjacent introns, and 3 kb of 5′ and 3′ flanking regions of the two genes (Supplementary Table S2). These primers were synthesized at the Beijing Genomics Institute (Beijing, China). DNA was extracted from each semen sample of 44 sires using a salting-out procedure, and the quantity and quality were measured by a NanoDrop 2000 spectrophotometer (Thermo Scientific, Hudson, DE, USA) and gel electrophoresis, respectively. We randomly mixed them into two pools used for all the polymerase chain reaction (PCR) amplifications with equal concentrations (50 ng/µL) for each DNA, and each pool included 22 sires. PCR amplifications for the two pooled DNA were performed in a final reaction volume of 25 µL, which consisted of 2 µL genomic DNA, 1.25 µL of each primer (10 mM), 12.5 µL Premix Taq^TM^ (Takara, Dalian, China), and 8 µL DNase/RNase-Free Deionized Water (Tiangen, Beijing, China). The amplification conditions were as follows: 5 min at 94 °C for initial denaturing followed by 35 cycles at 94 °C for 30 s, 60 °C for 30 s, 72 °C for 30 s and a final extension at 72 °C for 7 min. Then each PCR product was sequenced by ABI3730XL DNA analyzer (Applied Biosystems, Foster, CA, USA), and the sequences were aligned with the reference genome (UMD3.1.1) using the BLAST (https://blast.ncbi.nlm.nih.gov/Blast.cgi) to search the potential SNPs.

DNAs of the whole blood samples from 1065 Chinese Holstein cows were extracted using TIANamp Blood DNA Kit (Tiangen, Beijing, China) according to the manufacturer′s instructions. We measured the quantity and quality of the blood DNA using a NanoDrop 2000 spectrophotometer (Thermo Scientific, Hudson, DE, USA) and gel electrophoresis, respectively. The identified SNP were genotyped using the blood DNAs of these cows by the matrix-assisted laser desorption/ionization time of flight mass spectrometry assay (MALDI-TOF MS, Sequenom MassARRAY, Agena, San Diego, USA).

### 2.3. Estimation of linkage disequilibrium (LD)

We performed the extent of LD among the nine SNPs of *LIPK* and *LIPJ* identified in this study using the Haploview 4.1 (Broad Institute, Cambridge, MA, USA), and the haplotype blocks were estimated.

In addition, we also used the Haploview 4.1 to estimate the LD among the SNPs of *LIPK*, *LIPJ*, and *SCD* genes, including two in *LIPK*, seven in *LIPJ*, and 24 in *SCD* (Table S4). The SNPs (one in *LIPK*, one in *LIPJ*, and 24 in *SCD*) of the previous studies were not genotyped in this study. Therefore, we used the 1577 Holsteins with worldwide correlation from the 1000 Bull Genomes Project [22] to estimate the LD.

### 2.4. Association Analyses

For the association analyses between the identified SNPs or haplotype blocks and 24 milk FAs, 3335 individuals had their pedigree traced back to three generations. The SAS 9.2 mixed procedure was employed for the analyses based on the following model:

$$Y_{ijklm}=µ+G_{i}+h_{j}+l_{k}+a_{l}+b\times M_{m}+e_{ijklm}$$

in which, $Y_{ijklm}$ was the phenotypic value of each trait of per cow; µ was the overall mean; $G_{i}$ was the fixed effect corresponding to the genotype or haplotype combination; $h_{j}$ and $l_{k}$ were the fixed effects of the farm and stage of lactation, respectively; $a_{l}$ was the random polygenic effect; $M_{m}$ was the fixed effect of calving month; b was the regression coefficient of covariate M; and $e_{ijklm}$ was the random residual. In addition, the additive (*a*), dominance (*d*), and allele substitution (α) effects were estimated by the following equations [23]:

$\alpha =\frac{AA-BB}{2},\;d=AB-\frac{AA+BB}{2},\;and\;\alpha =\alpha +d\left(q-p\right)$, where AA, AB, and BB represented the least square mean of milk fatty acids corresponding to the genotypes, and *p* and *q* were the allele frequencies of A and B, respectively.

### 2.5. Protein Structure and Function Prediction

We used the NPSA SOPMA SERVER (https://npsa-prabi.ibcp.fr/cgi-bin/npsa_automat.pl?page=/NPSA/npsa_sopma.html) to predict the change of protein secondary structure caused by the missense mutation in the coding regions of genes, and the parameters included window width (17), similarity threshold (8), and number of states (4). In addition, we assessed whether the missense mutation altered the protein function using the SIFT (http://sift.bii.a-star.edu.sg/) and PROVEAN (http://sift.jcvi.org/index.php) software. The score thresholds of the SIFT and PROVEAN were 0.05 and −2.5, respectively. If the score was below the thresholds, the SNP was predicted as a “deleterious” variant.

### 2.6. Predication of Transcription Factors Binding Sites (TFBSs)

We used the Genomatix software (http://www.genomatix.de/cgi-bin/sessions/login.pl?s=77bfbe2f9849561b2b3e91c76f124365) to predict the binding sites of the transcription factors (TFs) caused by the SNPs in 5′ flanking and untranslated region (UTR; matrix similarity threshold, MST > 0.80). During the prediction, the matrix exhibited a high conservation at this position (Ci-value >60%).

### 2.7. Gene Expressions Assay of LIPK and LIPJ

To further detect the potential function of *LIPK* and *LIPJ*, we detected expression levels in eight types of tissue, including mammary, uterus, ovary, liver, spleen, heart, kidney, and lung. Three lactating Chinese Holstein cows were selected from the Sanyuanlvhe Dairy Farming Center (Beijing, China). Eight types of tissue were collected from each cow and then stored at liquid nitrogen.

We isolated the total RNAs from the eight types of tissue from each cow using Trizol reagent (Invitrogen, Carlsbad, CA, USA) according to the manufacturer′s instructions. Subsequently, we measured the quantity and quality of RNA with the NanoDrop 2000 spectrophotometer (Thermo Scientific, Hudson, DE, USA) and gel electrophoresis, respectively. We used PrimerScriptH RT reagent Kit (TaKaRa Biotechnology Co., Ltd., Dalian, China) for the reverse transcription. We conducted the quantitative real-time PCR (qRT-PCR) using SYBR green fluorescence (Roche, Penzberg, Germany) with a volume of 15 µL consisted of 2 µL template of cDNA, 0.375 µL of each primer (10 mM), 4.75 µL distilled water, and 7.5 µL SYBR Green Mixture. The PCR conditions were: Denaturation 95 °C for 10 min; amplification 45 cycles at 95 °C for 10 s, 58 °C for 10 s, and 72 °C for 10 s. The qRT-PCR primers of *LIPK*, *LIPJ*, and *GAPDH* are shown in Table S4. We performed all the measurements in triplicate and the relative gene expression was normalized by the *GAPDH* with 2^−ΔΔCt^ method [24].

## 3. Results

### 3.1. SNPs Identification

We identified two SNPs for *LIPK* gene, namely, rs110322221 in the 5′ UTR and rs42774527 in the exon 11 (Table 1). For *LIPJ* gene, seven SNPs were identified (Table 1), including rs41606812 in the 5′ flanking region, rs211373799 in the 5′ UTR, rs42107056 in the 3′ UTR, and four SNPs (rs42107122, ss158213049726, rs209219656, and rs42107114) in the 3′ flanking region. Among the total nine SNPs, rs42774527 was a missense mutation with the substitution of amino acid from threonine (ACA) to lysine (AAA), and ss158213049726 was first identified in this study.

### 3.2. Associations between SNPs/Haplotype Blocks and Milk FAs

The association analyses were performed to test the genetic effects of all the nine SNPs in *LIPK* and *LIPJ* genes on milk FA traits, and the summary and detailed results are shown in Table 1 and Table S5, respectively. For *LIPK* gene, both rs110322221 and rs42774527 were significantly associated with C6:0, C8:0, C10:0, C14:0, C20:0, and total index (*p* = < 1.00 × 10^−4^ ~ 4.88 × 10^−2^). The SNP, rs110322221, also had strong associations with SFA (*p* = 4.38 × 10^−2^) and SFA/UFA (*p* = 3.14 × 10^−2^). The SNP, rs42774527, was also significantly associated with C17:0, C17:1, C18:1cis-9, and C17index (*p* = < 1.00 × 10^−4^ ~ 3.76 × 10^−2^). However, these two SNPs of *LIPK* have no association with C11:0, C12:0, C13:0, C14:1, C15:0, C16:0, C16:1, C18:0, C18index, C14index, C16index, and UFA (*p* > 5.00 × 10^−2^).

Regarding the *LIPJ* gene (Table 1 and Table S5), SFAs (C6:0, C8:0, C10:0, C14:0, C17:0, C18:0, C20:0, and SFA) and UFAs (C17:1, C18:1cis-9, and UFA) were significantly associated with at least one SNP (*p* = < 1.00 × 10^−4^ ~ 3.73 × 10^−2^). The C14index, C16index, and total index were strongly associated with at least three SNPs (*p* = 1.80 × 10^−3^ ~ 4.36 × 10^−2^). For C11:0, C12:0, C13:0, C14:1, C15:0, C16:0, C16:1, C18index, C17index, and SFA/UFA, no significant associations (*p* > 5.00 × 10^−2^) were identified.

In addition, the additive (*a*), dominance (*d*), and allele substitution (α) effects of the SNPs identified in *LIPK* and *LIPJ* genes are shown in Table S6. The additive, dominance, and allele substitution effects of the nine SNPs were significantly associated with 19 milk FAs (C6:0, C8:0, C10:0, C12:0, C14:0, C14:1, C16:1, C17:0, C17:1, C18:0, C18:1cis-9, C20:0, C14index, C16index, C17index, SFA, UFA, SFA/UFA, and total index; *p* < 5.00 × 10^−2^). While, for the other five traits (C11:0, C13:0, C15:0, C16:0, and C18index), no significant genetic effects (*p* > 5.00 × 10^−2^) were observed.

**Figure 1:**
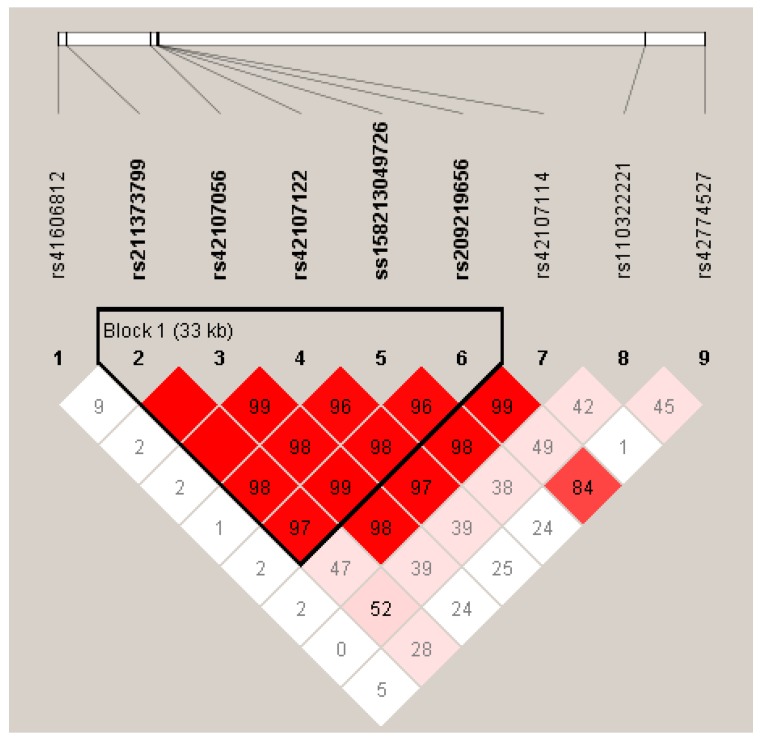
Linkage disequilibrium (LD) among the nine SNPs of *LIPK* and *LIPJ* genes (D′ = 0.96 ~ 1.00). D′ is the value of D prime between the two loci.

Furthermore, we estimated the LD among all the nine SNPs in *LIPK* and *LIPJ* genes, and discovered a haplotype block (D′ = 0.96 ~ 1.00; Figure 1), that only included five SNPs of *LIPJ* (rs211373799, rs42107056, rs42107122, ss158213049726 and rs209219656). There were four haplotypes (H1: CTCAG, H2: CCTGT, H3: ATCAG, and H4: CCTGG) in this haplotype block with the frequencies of 50.8%, 25.9%, 14.2%, and 7.6%, respectively. By using haplotype-based association analyses, we found that the haplotype block was significantly associated with nine milk FAs (C6:0, C8:0, C10:0, C17:1, C20:0, C14index, C17index, UFA, and total index; *p* = < 1.00 × 10^−4^ ~ 3.98 × 10^−2^) (Table 2).

### 3.3. Linkage disequilibrium among the SNPs of LIPK, LIPJ and SCD Genes

**Figure 2:**
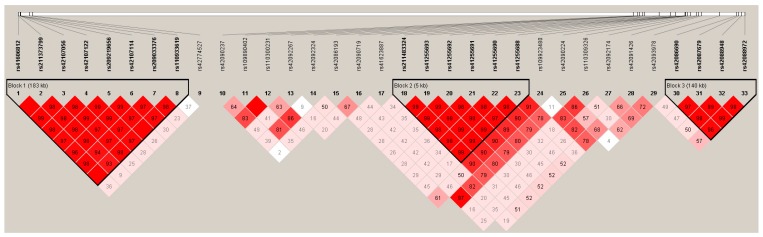
Linkage disequilibrium (LD) among the SNPs of *LIPK*, *LIPJ*, and *SCD* genes (D′ = 0.93 ~ 1.00). D′ is the value of D prime between the two loci. Haplotype block 1 included rs41606812, rs211373799, rs42107056, rs42107122, rs209219656, rs42107114, and rs209033376 of *LIPJ* and rs110933619 of *LIPK*. Haplotype block 2 and 3 included six (rs211483324, rs41255693, rs41255692, rs41255691, rs41255690, and rs41255688) and four SNPs (rs42086690, rs42087679, rs42088948, and rs42088972) of *SCD* gene, respectively.

To determine whether the genetic associations of *LIPK* and *LIPJ* on FAs were caused by the effect of *SCD*, we indirectly estimated the LD among the SNPs of *LIPK*, *LIPJ*, and *SCD* genes using the 1000 Bull data [22], and three haplotype blocks were observed (D′ = 0.93 ~ 1.00; Figure 2). The haplotype block 1 consisted of eight SNPs, including rs41606812, rs211373799, rs42107056, rs42107122, rs209219656, rs42107114, and rs209033376 of *LIPJ* and rs110933619 of *LIPK*. The haplotype block 2 and 3 were formed by six (rs211483324, rs41255693, rs41255692, rs41255691, rs41255690, and rs41255688) and four SNPs (rs42086690, rs42087679, rs42088948, and rs42088972) of *SCD* gene, respectively. The results indirectly showed that there was no linkage (r^2^ > 0.8) between *LIPK*/*LIPJ* and *SCD*, indicating that the genetic associations of *LIPK*/*LIPJ* with milk FAs were not due to the LD with *SCD.*

### 3.4. rs42774527 Caused the Changes of the LIPK Protein Structure and Function

We used the SOPMA software to predict the changes of the protein secondary structure for the missense mutation (rs42774527) in the exon 11 of *LIPK* gene, and found that the alpha helix was changed from 34.09% to 33.08%, extended strand from 19.95% to 19.44%, beta turn from 6.06% to 6.31%, and random coil from 39.90% to 41.16%. In addition, the SNP, rs42774527, was considered as a “deleterious” mutation by SIFT (score = 0.04) and PROVEAN (score = −3.135), that potentially altered the protein functions.

### 3.5. Transcription Factors Binding Sites (TFBSs) Changed by rs110322221 and rs211373799

**Figure 3:**
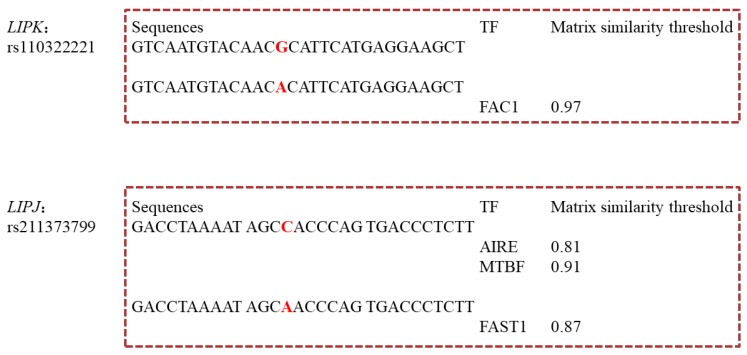
Changes of transcription factor binding site (TFBS) caused by the SNP in the 5′ untranslated region (UTR) of *LIPK* (Ci-value >60%). The SNPs in sequences are highlighted in red.

**Figure 4:**
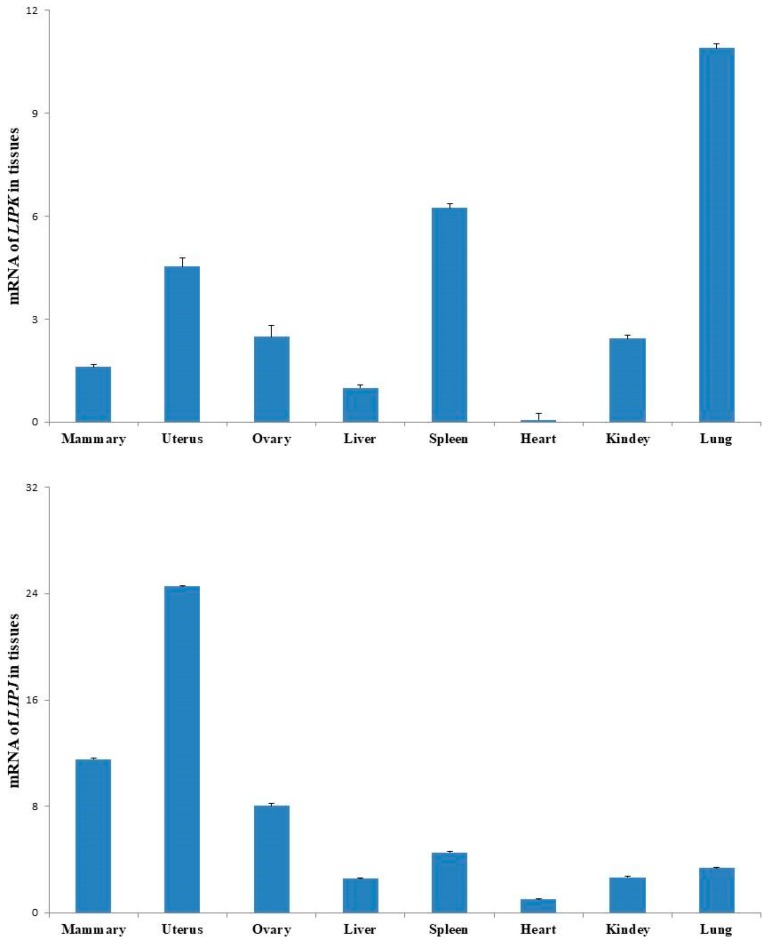
Relative mRNA expression of *LIPK* and *LIPJ* genes in eight tissues of lactating Holstein cows.

For the SNPs in 5′ flanking and UTR of *LIPK* and *LIPJ* genes, we predicted the changes of TFBSs caused by them. The allele A of rs110322221 in the 5′ UTR of *LIPK* gene was predicted to create the binding sites for the TF FAC1 (fetal alz-50 clone 1; MST = 0.97) (Figure 4). For the *LIPJ* gene, the allele C in the rs211373799 could create the binding sites for the TFs AIRE (autoimmune regulator; MST = 0.81) and MTBF (muscle-specific Mt binding site; MST = 0.91), and the allele A in this SNP could invent the TFBS for FAST1 (FAST-1SMAD interacting protein; MST = 0.87) (Figure 3).

### 3.6. Expressions of LIPK and LIPJ Genes

By using the qRT-PCR method, we observed that *LIPK* and *LIPJ* genes were expressed in eight types of tissue, including mammary, uterus, ovary, liver, spleen, heart, kidney, and lung. The relatively high expression levels of these two genes in the mammary gland implied that they might be involved in milk composition synthesis (Figure 4).

## 4. Discussion

Our previous GWAS identified that *LIPK* and *LIPJ* genes were both promising candidates for FAs in dairy cattle. In this study, we first demonstrated that both *LIPK* and *LIPJ* genes were mainly associated with saturated medium- and long-chain milk FAs.

SFAs are reported to increase the risks for diseases, while UFAs lower the risks [25,26]. C6:0 is related with the depressive symptoms phenotype [27]. C14:0 causes the nonalcoholic steatohepatitis associated with lipodystrophy [28]. C14:1 inhibits the formation of large multinucleated osteoclasts related with osteoporosis and other bone-lytic diseases [29]. C17:1 can be affected by the fatty acid two-hydroxylase to regulate intestinal homoeostasis in elegans [30]. C18:1cis-9 can reduce inflammation [31]. To date, no report has highlighted the correlation between *LIPK* and *LIPJ* and milk fatty acids in dairy cattle. Our association analysis results showed that genotypes AA of rs110322221 and AA of rs41606812 decreased milk contents of C8:0 and C20:0, respectively. Genotypes TT of rs42107122 deceased C14:0 compared with genotypes CC. Both the genotypes CC of rs42107056 and GG of ss158213049726 showed lower C14:0, and higher C17:1. Genotype TT of rs209219656, and haplotype H2 formed by five SNPs (rs211373799, rs42107056, rs42107122, ss158213049726 and rs209219656) increased C17:1. In the present study, we further verified that no LD (r^2^ > 0.8) was found between *LIPK*/*LIPJ* and *SCD*, implying that the associations of *LIPK* and *LIPJ* with milk FAs were not caused by the linkage to the *SCD*. In addition, we observed the relative high mRNA level of *LIPK* and *LIPJ* in the mammary gland, suggesting that the two genes might be related to the composition of milk fat. Furthermore, we observed the expressions of the two genes in the uterus, ovary, liver, spleen, heart, kidney, and lung. There has been no report about the expression of *LIPK* and *LIPJ* in these tissues until now. A recent study showed the *LIPK* expression level in granular keratinocytes of human abdominal skin, and suggested that the gene might play a highly specific role in the last step of keratinocyte differentiation to regulate the lipid metabolism of the most differentiated epidermal layers [32]. We hypothesize that *LIPK* and *LIPJ* could play roles in some biological functions specific for these types of tissue. Considering the already identified significant genetic effects of *LIPK* and *LIPJ* on milk fatty acids, the six SNPs, including rs110322221 of *LIPK*, rs41606812, rs42107056, rs42107122, ss158213049726, and rs209219656 of *LIPJ*, could be used as molecular markers for genetic improvement programs of dairy cattle through marker-assisted selection to provide the market with better milk with high UFA and low SFA contents.

SNP, rs42774527, as a missense mutation, was predicted to alter the protein secondary structure, and was a “deleterious” mutation changing protein functions. The association analysis showed that the cows with the AA genotype of rs42774527 had significantly higher C14:0, and lower C6:0, C17:0, C18:1cis-9, and total index than those with CC genotype. Hence, the changes in LIPK protein’s secondary structure might impact the LIPK protein’s ability to form milk FA traits.

Two SNPs, rs110322221 of *LIPK* and rs211373799 of *LIPJ*, in their 5′ UTR created the TFBSs for FAC1, AIRE, MTBF, or FAST1. The association analysis showed that the cows with the genotype AA of rs110322221 had significant lower C8:0 than those with genotype GG, and the genotype CC of rs211373799 significantly lowered the C6:0 and C8:0, and increased the C20:0, than the genotype AA. We didn’t find any reports about the regulation of TFs, FAC1, AIRE, MTBF, and FAST1 on lipid metabolism in the mammary gland. Previous studies showed that FAC1 represses transcription of the *APP* gene to influence neuronal development and neuro-degenerative diseases in human [33], AIRE transcriptionally activates the *IL-6* in androgen-independent cells to modulate the prostate tumor microenvironment in mice [34], and FAST1 directly regulates mesodermal gene expression to mediate the form of mesodermal in the Xenopus embryo [35]. We herein deduced that FAC1, AIRE, MTBF, and FAST1 could up-/down-regulate the expression of *LIPK* or *LIPJ* to affect the milk FA traits.

There was a limitation in this study. We used DNA pooling to search SNPs, which may have resulted in rare SNPs with very low allele frequencies being missed. To identify as many possible variants in our population as possible, we randomly pooled the 44 sires into two pools in order to ensure the concentration (50 ng/µL) for each sire was high enough for the identification of potential SNPs. Moreover, we also have performed PCR product sequencing based on pooled DNA to identify SNPs in several previous studies [36,37,38,39,40,41], and we found that this method can detect the SNP with a low frequency of 0.0419. In addition, as for the variant with a very low frequency, the smaller number of individuals with the homozygous genotype of rare alleles could impact the statistical power of the association analysis result.

## 5. Conclusions

Our initial GWAS identified *LIPK* and *LIPJ* genes as two promising candidates for FAs in dairy cattle. In the study, we further demonstrated that both *LIPK* and *LIPJ* showed significant associations with milk FA traits in the Chinese Holstein population. These SNPs, including rs110322221 of *LIPK*, rs41606812, rs42107056, rs42107122, ss158213049726, and rs209219656 of *LIPJ*, and the haplotype H2 could be used as genetic markers to reduce the milk SFA or increase milk UFA.

## Figures and Tables

**Table 1:** Summary of the detailed information and association analysis results of nine identified single nucleotide polymorphisms (SNPs).

Gene	SNP Name	Location	Position	GenBank no.	Significant Milk FAs	*p*-Interval	Allele	TFBS	Amino	Changes of Protein Secondary Structure				SIFT	PROVEAN
			(UMD 3.1)						Acid	Alpha Helix	Extended Strand	Beta Turn	Random Coil		
*LIPK*	g.10428101G>A	5′ UTR	Chr26: 10428101	rs110322221	C6:0, C8:0, C10:0, C14:0, C20:0, SFA, SFA/UFA, total index	<1.00 × 10^−4^~4.88 × 10^−2^	G								
							A	FAC1							
	g.10449831C>A	Exon-11	Chr26: 10449831	rs42774527	C6:0, C8:0, C10:0, C14:0, C17:0, C17:1, C18:1cis-9, C20:0, C17index, total index	<1.00 × 10^−4^~3.76 × 10^−2^	C		Thr	34.09%	19.95%	6.06%	39.90%	0.04	-3.315
							A		Lys	33.08%	19.44%	6.31%	41.16%		
*LIPJ*	g.10214117A>C	5′ flanking region	Chr26: 10214117	rs41606812	C17:0, C17:1, C20:0	<1.00 × 10^−4^~2.55 × 10^−2^	A								
							C								
	g.10217380C>A	5′ UTR	Chr26: 10217380	rs211373799	C6:0, C8:0, C10:0, C14:0, C17:1, C20:0	<1.00 × 10^−4^~3.41 × 10^−2^	C	AIRE							
								MTBF							
							A	FAST1							
	g.10247997T>C	3′ UTR	Chr26: 10247997	rs42107056	C6:0, C8:0, C14:0, C17:1, C14index, C16index, SFA, total index	1.30 × 10^−3^~2.15 × 10^−2^	T								
							C								
	g.10250098C>T	3′ flanking region	Chr26: 10250098	rs42107122	C6:0, C14:0, C14index, C16index, SFA, UFA, total index	3.00 × 10^−4^~4.36 × 10^−2^	C								
							T								
	g.10250120A>G	3′ flanking region	Chr26: 10250120	ss158213049726	C6:0, C10:0, C14:0, C17:1, C18:1cis-9, C14index, C16index, SFA, UFA, total index	<1.00 × 10^−4^~3.37 × 10^−2^	A								
							G								
	g.10251075G>T	3′ flanking region	Chr26: 10251075	rs209219656	C6:0, C14:0, C17:1, C20:0, C14index, C16index	1.00 × 10^−3^~3.68 × 10^−2^	G								
							T								
	g.10251111T>C	3′ flanking region	Chr26: 10251111	rs42107114	C6:0, C8:0, C10:0, C17:1, C20:0, C14index, total index	<1.00 × 10^−4^~4.22 × 10^−2^	T								
							C								

Notes: Notes: UTR: Untranslated Regions. *p* referred to the significances of the association analysis between each SNP and milk fatty acid traits. *p* was the raw value. We considered the significance of *p* < 5.00 × 10^−2^. TFBS: changes of the transcription factor binding site by the SNP. FAC1: fetal alz-50 clone 1. AIRE: autoimmune regulator. MTBF: muscle-specific Mt binding site. FAST1: FAST-1SMAD interacting protein. The mutation is considered a “deleterious” mutation for protein function by the prediction using SIFT (threshold of 0.05) and PROVEAV (threshold of −2.5) softwares. Thr: threonine (ACA). Lys: lysine (AAA). The SNP, ss158213049726, was first identified in this study.

**Table 2:** Associations of the haplotype block among five SNPs in *LIPJ* gene with milk fatty acids traits in dairy cattle (Least square mean ± Standard error).

Haplotype Combination (No.)	C6:0 (%)	C8:0 (%)	C10:0 (%)	C11:0 (%)	C12:0 (%)	C13:0 (%)	C14:0 (%)	C14:1 (%)	C15:0 (%)	C16:0 (%)	C16:1 (%)	C17:0 (%)
H1H1 (236–250)	0.4915 ± 0.0138 ^A^	0.9582 ± 0.0120 ^Aa^	2.8777 ± 0.0356 ^Aa^	0.0607 ± 0.0028	3.0778 ± 0.0464	0.1002 ± 0.0035	10.3703 ± 0.0785	0.6266 ± 0.0209	0.9980 ± 0.0143	34.8257 ± 0.2024	1.2791 ± 0.0283	0.5652 ± 0.0036
H1H2 (196–215)	0.5079 ± 0.0143 ^A^	0.9214 ± 0.0126 ^Ab^	2.8373 ± 0.0368 ^A^	0.0607 ± 0.0029	3.0641 ± 0.0478	0.1020 ± 0.0036	10.2305 ± 0.0814	0.6608 ± 0.0219	0.9895 ± 0.0150	34.5606 ± 0.2108	1.3468 ± 0.0295	0.5725 ± 0.0038
H1H3 (134–147)	0.4066 ± 0.0163 ^B^	0.8758 ± 0.0139^B^	2.6856 ± 0.0408 ^B^	0.0536 ± 0.0032	2.9704 ± 0.0526	0.0971 ± 0.0042	10.3702 ± 0.0897	0.6668 ± 0.0249	1.0024 ± 0.0170	34.9864 ± 0.2406	1.3309 ± 0.0333	0.5729 ± 0.0043
H1H4 (84–91)	0.4642 ± 0.0186 ^AB^	0.9134 ± 0.0159 ^ABb^	2.7806 ± 0.0463	0.0593 ± 0.0038	2.9892 ± 0.0611	0.0981 ± 0.0050	10.3107 ± 0.1029	0.6756 ± 0.0291	0.9988 ± 0.0200	34.7553 ± 0.2768	1.3364 ± 0.0384	0.5688 ± 0.0050
H2H2 (94–99)	0.5145 ± 0.0180 ^ACa^	0.9540 ± 0.0154 ^A^	2.7562 ± 0.0451 ^ABb^	0.0590 ± 0.0037	3.0134 ± 0.0584	0.0978 ± 0.0049	10.2163 ± 0.0996	0.6841 ± 0.0285	0.9919 ± 0.0192	34.7388 ± 0.2684	1.3455 ± 0.0376	0.5670 ± 0.0049
H2H3 (52–56)	0.5886 ± 0.0215 ^Cb^	0.9553 ± 0.0182^A^	2.7167 ± 0.0526 ^ABb^	0.0551 ± 0.0044	2.9365 ± 0.0691	0.1012 ± 0.0059	10.1683 ± 0.1181	0.6606 ± 0.0340	0.9837 ± 0.0236	34.8336 ± 0.3231	1.3490 ± 0.0443	0.5638 ± 0.0059
*p*	<1.00 × 10^−4 **^	<1.00 × 10^−4 **^	<1.00 × 10^−4 **^	2.50 × 10^−1^	8.57 × 10^−2^	9.06 × 10^−1^	1.74 × 10^−1^	2.91 × 10^−1^	9.65 × 10^−1^	6.11 × 10^−1^	1.75 × 10^−1^	2.64 × 10^−1^
Haplotype combination (No.)	C17:1 (%)	C18:0 (%)	C18:1cis-9 (%)	C18index (%)	C20:0 (%)	C14index (%)	C16index (%)	C17index (%)	SFA (%)	UFA (%)	SFA/UFA (%)	Total index (%)
H1H1 (212–250)	0.1877 ± 0.0028^A^	14.0724 ± 0.1029	19.0132 ± 0.1380	57.1817 ± 0.3145	0.1671 ± 0.0020^a^	5.7655 ± 0.1594 ^Aa^	3.5534 ± 0.0726	24.5170 ± 0.2489^a^	68.2270 ± 0.1876	30.1878 ± 0.1709	2.3037 ± 0.0250	27.2671 ± 0.1630^a^
H1H2 (174–215)	0.1955 ± 0.0029^a^	14.0472 ± 0.1081	19.1074 ± 0.1447	57.2138 ± 0.3281	0.1737 ± 0.0021 ^Ab^	6.1702 ± 0.1694	3.7593 ± 0.0752	25.3334 ± 0.2578 ^b^	68.0475 ± 0.1961	30.3502 ± 0.1793	2.2650 ± 0.0260	27.5520 ± 0.1695
H1H3 (119–148)	0.1944 ± 0.0033	14.0278 ± 0.1234	18.9345 ± 0.1647	56.9697 ± 0.3745	0.1696 ± 0.0024	6.1980 ± 0.1838	3.6901 ± 0.0849	24.8310 ± 0.2861	68.0476 ± 0.2240	30.3144 ± 0.2052	2.2995 ± 0.0298	27.2367 ± 0.1857 ^a^
H1H4 (79–91)	0.1960 ± 0.0039	14.1424 ± 0.1452	19.4779 ± 0.1965	57.8644 ± 0.4411	0.1619 ± 0.0028 ^B^	6.3962 ± 0.2167 ^b^	3.7332 ± 0.0982	25.1307 ± 0.3321	67.5861 ± 0.2651	30.7677 ± 0.2399	2.2465 ± 0.0350	27.9861 ± 0.2200 ^b^
H2H2 (86–99)	0.2014 ± 0.0037 ^B^	13.8249 ± 0.1418	19.3393 ± 0.1880	57.8175 ± 0.4261	0.1680 ± 0.0027	6.5399 ± 0.2096	3.7366 ± 0.0958	25.2834 ± 0.3248	67.5006 ± 0.2558	30.8790 ± 0.2348	2.2421 ± 0.0337	27.9063 ± 0.2131 ^b^
H2H3 (41–56)	0.1822 ± 0.0045 ^Ab^	13.9019 ± 0.1738	19.0366 ± 0.2300	57.2853 ± 0.5135	0.1605 ± 0.0035 ^B^	6.1413 ± 0.2510 ^B^	3.7223 ± 0.1123	24.1729 ± 0.3870 ^a^	67.9823 ± 0.3101	30.4244 ± 0.2834	2.2738 ± 0.0414	27.5623 ± 0.2559
*p*	2.00 × 10^−4 **^	4.92 × 10^−1^	1.05 × 10^−1^	3.13 × 10^−1^	<1.00 × 10^−4 **^	1.90 × 10^−3 **^	7.34 × 10^−2^	2.40 × 10^−3 **^	5.06 × 10^−2^	3.98 × 10^−2 *^	3.32 × 10^−1^	9.00 × 10^−4 **^

Notes: *p* referred to the significance of the association analysis between each haplotype block and milk fatty acid traits. *p* was the raw value. * indicated *p* < 5.00 × 10^−2^. ** indicated *p* < 1.00 × 10^−2^. Different letter (small letters: *p* < 5.00 × 10^−2^; capital letters: *p* < 1.00 × 10^−2^) superscripts indicated significant differences among the haplotype combinations. The number in the brackets represented the number of cows for the corresponding haplotype combination.

## Supplementary Materials

The following are available online at http://www.mdpi.com/2073-4425/10/2/86/s1, Table S1: Summary of the phenotype values. Table S2: PCR primers of *LIPK* and *LIPJ* genes. Table S3: SNPs used for the LD analysis between *LIPK*, *LIPJ*, and *SCD*. Table S4: Primers used for the amplification of bovine *LIPK*, *LIPJ*, and *GAPDH* genes by quantitative real-time PCR. Table S5: Associations of nine SNPs of *LIPK*, and *LIPJ* genes with fatty acid traits in dairy cattle (Least square mean ± Standard error). Table S6: The additive (a), dominant (d), and allele substitution (α) effects of nine SNPs on milk fatty acid traits in dairy cattle.
